# Passion for Life: Lived Experiences of Patients after Coronary Artery Bypass Graft

**Published:** 2015-07-03

**Authors:** Nooredin Mohammadi, Mohammad Abbasi, Alireza Nikbakht Nasrabadi, Abbas Salehiomran, Saeid Davaran, Reza Norouzadeh

**Affiliations:** 1 *Department of Critical Care Nursing, School of Nursing and Midwifery, Iran University of Medical Sciences, Center for Nursing Care Research, Tehran, Iran.*; 2 *International Campus, Iran University of Medical Sciences, Tehran, Iran.*; 3 *Tehran University of Medical Sciences, Tehran, Iran.*; 4 *Shahed University, Nursing and Midwifery Faculty, Tehran, Iran.*

**Keywords:** *Coronary artery bypass graft surgery*, *Hermeneutics*, *Qualitative study*

## Abstract

**Background:** Coronary artery bypass graft surgery (CABG) improves the quality of life, increases survival, and influences the patient's mental and emotional aspects. Little information is available on the lived experience of Iranian patients after this surgery. Understanding the lived experiences of patients will help health professionals with better provision of high quality care.

**Methods:** This hermeneutic phenomenological study aimed to understand the lived experience of patients after CABG. Van Manen's method was used to conduct the study. A semi-structured, face-to-face interview technique was employed to explore the experiences of the patients following surgery. Seven men and 4 women between 49 and 80 years old were interviewed.

**Results:** Passion for life was the main theme extracted from the participants’ interviews. This theme comprised the three sub-themes of receiving attention from family, being hopeful, and being spiritually oriented.

**Conclusion: ** The results showed that the participants experienced passion for life after their surgery. This finding reveals that patients tend to find a new perspective on life and their health after surgery.

## Introduction

Coronary artery disease (CAD) is one of the most common health problems and results in high mortality and morbidity in developed and developing countries.^1^ Also in Iran, 45% of deaths are estimated to be in consequence of CAD.^        2 ^ CAD usually leads to the exacerbation of angina, dyspnea, fatigue, and physical condition. These problems reduce the patient's ability to perform daily activities.^3^ The progression of CAD renders the use of coronary artery bypass graft surgery (CABG) inevitable. Indeed, CABG is considered as one of the most effective method for the control and treatment of CAD.^1^ The main purpose of this intervention is to restore cardiac perfusion and reduce the mortality and morbidity due to ischemic heart disease. CABG can improve the quality of life and increase survival.^4^ In addition, this surgical modality has a significant impact on the patient's mental and emotional aspects.^5^ There are several studies regarding the outcomes of CABG and its effect on the quality of life. 1  Nevertheless, there is a dearth of data in the existing literature on the lived experience of patients after this surgery.^6^

Several studies have explained the lived experience of patients after CABG from a cultural perspective.^7^^, ^8 Basically, culture influences how patients interpret health and illness in an effort to understand their own illness and pain.^     9 ^ Culture can be a powerful force to modify individuals’ behavior.^10^ Culture also impacts health and the capacity of patients to adhere to their treatments. ^11^ Therefore, cultural beliefs influence health-related behavior in all societies with different cultural backgrounds.^   12 ^ For example, people from the Jehovah’s Witness culture refuse to have blood transfusion. ^13^ The Iranian culture is an amalgamation of Persian and Islamic cultures;^14^ thus, values and beliefs shared by Iranians are influenced by Islam. In the Iranian culture and similar ones, family is the foundation of the human society and civility. ^15^ Family is believed to play a crucial role in the provision of a healthy life, particularly mental health.^16^ Also of great significance is the part which family plays in supporting its members. When there is a sick person in the family, the family members assume different roles to provide physical, emotional, and financial support.^17^ Therefore, as regards CABG, it would be safe to assume that family exerts a great influence on the experience of patients following this surgical modality.^18^ What the existing literature lacks in this regard, however, is research on the lived experience of patients after CABG in Iran. Understanding the lived experience of post-CABG patients would help health care professionals to devise more appropriate plans for self-care behaviors and adherence to the treatment plan.

This article is part of a larger hermeneutic phenomenological inquiry, which aimed to explore a deeper understanding of the lived experience of patients after CABG. Accordingly, the present study will discuss the findings on the lived experience of post-CABG patients and the role which family plays in their lived experience. 

## Methods

Phenomenological research based on Heidegger’s beliefs is known as hermeneutic or interpretive research. The objective of this type of research is to understand the concept of human experiences (Cohen and Omery, 1994). Walters (1995) adds that in a hermeneutic research, the researcher is an active participant in the process of interpretation rather than a passive receiver of information. Van Manen believes that information should be extracted by analyzing the reflective descriptions of people with related experience and suggests that nursing researchers find a better understanding of patients by studying their life experiences. Table 1 summarizes the six activities suggested by Van Manen used in the present study.^19^

**Table 1:** Summary of van Manen’s methodological activities^19^

Van Manen’s Methodological Activities	Researchers’ Activities
Turning to the nature of lived experience	Thinking about living with CABG and asking what experience the patients have after surgery to develop the phenomenological inquiry
Investigating experience as we live it	Contemplating on the Iranian culture and the perspective of Iranian people on health and illness, having a prolonged engagement with post-CABG patients, and conducting in-depth interviews
Reflecting on essential themes	Listening to audio tapes, reading transcripts, immersing oneself in the data, and conducting thematic data analysis
Hermeneutic phenomenological writing	Writing about emerged themes and sub-themes and creating an in-depth phenomenological text
Maintaining a strong and oriented nursing relation to the phenomenon	Discussing the themes in relation to lived experience
Balancing the research context by considering parts and whole	Moving between transcripts and themes in relation to lived experience following CABG

Six methodical activities proposed by van Manen (1990)

Adapted from: Van Manen M, ed. Researching Lived Experience: Human Science for an Action Sensitive Pedagogy. New York: University of Western Ontario; 1990.

**Table 2:** Demographic characteristics of the study participants

Participant (Number)	Age (y)	Sex	Marital status	Occupation	Time after CABG (y)
1	54	male	married	Employee	4
2	62	male	married	Retired	13
3	57	female	married	Housekeeper	5
4	80	male	married	Retired	2
5	53	male	married	Self-employed	2
6	50	female	married	Housekeeper	1
7	68	male	married	Retired	3
8	61	male	married	Self-employed	3
9	49	female	married	Housekeeper	3
10	54	female	married	Housekeeper	5
11	61	male	married	Retired	2

CABG, Coronary Artery Bypass Graft

A purposeful sampling method was used to recruit the eligible participants from the outpatient clinic in two heart centers, Tehran, Iran, where the participants underwent routine check-ups. The sampling period was from June 2012 to July 2013. Eligible participants were patients having undergone first-time CABG in the preceding 6 months. Ability to communicate in Farsi was another criterion. Participants with a history of psychological problems were excluded from the study.

The first researcher met the potential participants and assessed their eligibility for recruitment in the study. Then the aim of the study was explained to the participants. Before conducting the interview, the first researcher had an introductory meeting with the participants. The goal of the introductory meeting was to establish trust between the participants and the researcher. The first researcher explained the goal of the study to the participants and answered their queries. In this study, a semi-structured, face-to-face interview technique was employed to collect data. The interviews were conducted in Farsi in a quiet room in the clinic. The duration of the interviews was from 55 to 70 minutes. All the interviews were audio taped. 

Data collection was continued until saturation was achieved. Saturation was defined as the time when data collection showed no information. Then all the interviews were transcribed verbatim. The transcript of each interview was typed.

 Data analysis was performed using the detailed, selective, and holistic approach of thematic analysis.^19^ For this purpose, the researcher studied the text several times to achieve a comprehensive understanding of the participants' experience. Words, phrases, and sentences were extracted according to the main theme of the study, i.e. the lived experience of patients after CABG. 

The rigor of study was achieved through the following strategies. An effective trust-based relationship was established between the participants and the researchers before conducting the interviews. Data from the interviews were presented to the participants after analysis, and their reflections were considered in the process of data analysis. The extracted themes were discussed with most of the participants for their approval. Furthermore, every step of the study was checked with supervisors and experts to receive their recommendations in order to ensure the accuracy and appropriateness of the study process. 

This study was approved by the Ethics Committee of Tehran University of Medical Sciences. Before the interview, the investigator oriented each participant to the study and obtained a written informed consent. The participants knew that they had the freedom to refuse to continue at any time during the study and were given reassurances that the results would be confidential and would only be used for academic purposes. The investigator was in contact with the participants by e-mail and telephone calls, and the subjects were informed about the results of the research. 

## Results

The participants were 7 men and 4 women between 49 and 80 years of age (Table 2). All the participants were married and lived with their own families. The theme “passion for life” was one of the extracted themes from the participants’ lived experience. Passion for life consisted of the three sub-themes of receiving attention from family, being hopeful, and being spiritually oriented. The participants expressed the feeling that their life after CABG was more meaningful and that they looked forward to having a better and longer life. A participant stated, "My life has become warmer since the surgery, and I want to live more." All the participants pointed out that their positive attitude toward life and family encouraged them to look forward to a longer life.

***Receiving attention from family***

In the present study, the subjects expressed the feeling that their family members were a great source of support, sympathy, and help during their hospitalization and after surgery. The attention which the participants received from family in this critical stage was cited as the most common source of motivation to reflect more on the significance of family. This theme reveals how the participants had been supported both physically and psychologically by family members, imbuing them with encouragement, reassurance, and peace of mind. Receiving attention from family led to passion for life following cardiac surgery in our study population. 

A participant remarked, "My children and wife encouraged me to have surgery. They constantly told me that I should undergo surgery. So, I had the surgery because they loved me and wanted me to live with them longer. After surgery, I realized how supportive they were. That’s why I feel myself much closer to them and our relationship is much better." Another participant said, "After heart surgery, my family members were around me and helped me. To be honest, until then, I hadn't realized how important a person I am in the family." 

*Being h* *opeful*

Our study population had consented to undergo CABG hoping to resume a relatively healthy and normal life. After surgery, they observed an ongoing improvement in their physical and mental status, which increased their hope for a longer life. A participant remarked, "Since the surgery, I have improved every day. When I think that my heart problems have been resolved, I feel more hopeful about the future." Another participant said, "I had a small kid; I was sure that God would give me the chance to return into the bosom of the family."

*Being spiritually oriented*

The participants of the present study reported that spirituality was the most important factor enabling them to recover after surgery. For the participants, spirituality was a crucial aspect of life because they believed that God had gifted them a new lease on life. Spirituality was also an important factor in our study population's passion for life after surgery. A participant declared, "My religious beliefs helped me a great deal to cope with the surgery. Allah says in the Holy Quran, 'Verily hearts are calm in the remembrance of Allah'." Another participant remarked, "I pray to God to express my gratitude for his having saved my life." One of the participants stated, "I think my religious beliefs saved my life. I was recalling Quran verses and was praying in my head before, during, and after surgery. I think my religious beliefs have grown much stronger since the surgery.” Another participant said, “I am saying that I didn’t believe in God, but you know, my attention to my religious mission is more serious.”

## Discussion

The results of the present study showed that the participants felt passion for life following CABG thanks to receiving attention from family, being hopeful, and being spiritually oriented. Family is known to play a vital role in the care and recovery process of a patient. 20 The participants in the current study pointed out that their family members were always supportive after surgery. They had a sense of security and morale boost with their own family compeers. Navab et al. noted that altruism and strong family ties are the two basic characteristics of the Iranian culture and that Iranians are highly committed to maintaining close relationships with their family and providing care for a sick family member.^21^

In the current study, the participants were hopeful and optimistic about life and the future because of improvement in their condition and feeling of trust. Ahmad et al. believed that family support provides not only physical and emotional comfort but also hope and a sense of belonging in the patient. Karlsson et al. reported hopelessness 6 months after cardiac surgery in patients with poor family support.^22^ For the participants of the study, surgery was a source of hope and a feeling of well-being. Likewise, the results of another published study indicate that surgery is associated with hope among both patients and their families.^23^

Our results highlight the role of spirituality in the study population's passion for life after cardiac surgery. All the participants underscored the importance of spirituality in their life after surgery, stating that reciting the Quran and recalling God during the pre- and postoperative periods further nurtured their spiritual aspect. Spirituality can be portrayed as a source of inner peace, comfort, and emotional strength.^24^ Koenig et al. pointed out that individuals with stronger religious beliefs are liable to have better adaptation with difficult life situations such as cardiac surgery.^25^ Rassool et al. reported a relationship between spirituality and recovery.^26^ Ebadi et al. also described the psychology of religious belief in helping people to cope with their life events, reporting that spirituality can create a feeling of hope, comfort, and emotional peace as well as closeness to others, opportunity for self-actualization, and intimacy with God.^27^

## Conclusion

The results of the present study demonstrated that the participants experienced passion for life after undergoing CABG. Passion for life enabled them to better appreciate the role of family, to be more hopeful, and to be more spiritually oriented. Our findings of the lived experience of post-cardiac surgery patients could be useful to health professionals and, in particular, nurses in their provision of care to this group of patients. 

## Notes:

***This paper should be cited as:*** Mohammadi N, Abbasi M, Nikbakht Nasrabadi A, Salehiomran A, Davaran S, Norouzadeh R. Passion for Life: the Experiences of Patients after Coronary Artery Bypass Graft. J Teh Univ Heart Ctr 2015;10(3):129-133.
